# Public Support for the Imposition of a Tax on Sugar-Sweetened Beverages and the Determinants of Such Support in Spain

**DOI:** 10.3390/ijerph19073758

**Published:** 2022-03-22

**Authors:** Sara Fernández Sánchez-Escalonilla, Carlos Fernández-Escobar, Miguel Ángel Royo-Bordonada

**Affiliations:** 1 Department of Preventive Medicine and Public Health, Albacete University Teaching Hospital Complex, 02006 Albacete, Spain; 2 Public Health and Epidemiology Research Group, Faculty of Medicine and Health Sciences, University of Alcalá, 28871 Alcalá de Henares, Spain; carlos.fernandezesco@edu.uah.es; 3 National School of Health, Carlos III Institute of Health, 28029 Madrid, Spain; mroyo@isciii.es

**Keywords:** tax, sugar-sweetened beverages, policies, obesity, interviews, Spain

## Abstract

(1) Background: Taxes on sugar-sweetened beverages are an effective public health intervention, but can be difficult to implement in the absence of public support. This is the first study to analyze the Spanish population’s support for a tax on sugar-sweetened beverages. (2) Methods: We conducted a cross-sectional study of a representative sample of the Spanish adult population (*n* = 1002), using a computer-aided telephone interview with a questionnaire on nutritional policies. The support for the tax was calculated by the percentage of those who agreed plus those who strongly agreed with the measure. The sociodemographic determinants of support for the tax were analyzed using chi-squared test (χ^2^) and Poisson multiple regression models with robust variance. (3) Results: Of the participants, 66.9% supported a tax on sugar-sweetened beverages. Support for the tax was 9.2% higher (70% vs. 64.1%) when responders were first asked about support for tax relief and subsidies for healthy foods (*p* = 0.049). Support for the tax was 16% and 35% lower among persons reporting center and right-wing political sympathies (*p* < 0.01), and 16% lower among regular consumers of sugar-sweetened beverages (*p* = 0.01). (4) Conclusions: A clear majority of the Spanish population is in favor of imposing a tax on sugar-sweetened beverages. Awareness-raising campaigns and a policy of combining the measure with subsidies or tax cuts on healthy foods could increase the level of support among those currently against the intervention.

## 1. Introduction

Intake of sugar-sweetened beverages increases the risks of obesity [1], diabetes [2], cardiovascular disease [3], cancer [4], and all-cause mortality [5]. In 2016, the World Health Organization (WHO) recommended the imposition of a tax on sugar-sweetened beverages of at least 20% of the retail price, with the aim of reducing their negative impact on health. This measure has shown itself to be effective in reducing the purchase and consumption of sugar-sweetened beverages [6], thus serving to sensitize the population to the health risks entailed in consuming such drinks.

The Spanish adult population consumes a mean of 246 mL/day of sugar-sweetened beverages, including soft drinks, fruit juices, and fruit drinks [7]. This consumption is higher among adolescents, rising to 450 mL/day [8] and providing more than 6% of their total caloric intake [9], thereby contributing to the high prevalence of obesity in Spain [10]. The intake of sugar-sweetened beverages is higher in Spanish children and adolescents than in those from other European countries, such as France and Belgium [8], where obesity prevalence is lower than in Spain [11]. Southern European countries, including Spain, have the highest prevalence of obesity and severe obesity among children [12,13]. In 2016, the Minister of Inland Revenue therefore announced the establishment of a tax on sugar-sweetened beverages in Spain, but the Government withdrew the proposal in the face of pressure from the soft drink industry and sugar beet sector [14]. One year later, the Catalonian Regional Authority imposed a specific tax on sugar-sweetened beverages for health reasons, subject to the requirement that the full burden of this levy was to be borne by the end consumer [15].

The most common soft drinks in the Spanish market have an amount of sugar that ranges between 78 g and 54 g per 600 mL [16]. The average amount of soft drink consumed in Spain during the year 2019 according to the report of the Ministry of Agriculture, Fisheries and Food per person and year was 38.85 L [17]. In recent years, the consumption of fruit drinks with and without gas has decreased but the consumption of other sugar-sweetened beverages such as energy drinks has increased [18].

In Spain, the Law on Food Safety and Nutrition establishes that the sale of foods and beverages with a high content of saturated fatty acids, trans fatty acids, salt, and sugars is not allowed in schools [19], but the regulation that will determine the allowed contents has not been developed yet. Currently, only a guide with voluntary recommendations is available [20], and compliance with its criteria is very low [21].

Evidence to date shows that acute exposure to unhealthy food or nonalcoholic beverage advertising on television or the Internet increases the intake of those products, especially in children [22,23]. Therefore, States have the responsibility to regulate advertising to protect children from exposure to the marketing of ultraprocessed foods and beverages [24].

When it comes to drawing up public health policies, an important factor to be taken into consideration is their possible acceptance by the population [25], particularly when personal freedoms and individual responsibility are invoked to oppose them [26]. In the absence of public support, even the best-intentioned and most carefully drawn up policy can prove difficult to pass into law or implement [27,28]. Interviews conducted in a number of countries have detected a varying degree of popular support for the imposition of taxes on sugar-sweetened beverages in around 50% of the population, linked to sociodemographic factors and the attribution of obesity to different causes [27,29,30,31,32,33,34,35,36,37,38].

In Spain, while the levying of a tax on sugar-sweetened beverages enjoys the support of scientific associations, health professionals, and civil society organizations [39], to our knowledge there are no data available on the general population’s opinion on the matter. Hence, this study aimed to describe the degree to which the Spanish population supports the introduction of a tax on sugar-sweetened beverages, and analyze the determinants of such support. Furthermore, it also analyzed the individual and environmental factors related to the consumption of the sugar-sweetened drinks to which obesity is attributed.

## 2. Materials and Methods

### 2.1. Study Design and Participants

We conducted a cross-sectional study by interviewing persons aged 18 years and over, resident in Spain. As its base, the initial sampling framework took homes in Spain having a fixed telephone installed in September 2018. A total of 99.6% of homes had a telephone; of these, 23.9% only had a mobile telephone, 1.6% only had a fixed telephone, and 74.2% had both [40]. To extend the study’s coverage to persons who did not possess a fixed telephone or whose names had not been recorded in the database at their own request, a mobile telephone database was incorporated into the sampling framework, establishing a 50–50 distribution between fixed and mobile numbers. The mobile telephone database was created with randomly generated numbers starting with 6 and 7, deleting the prefixes (the first 3 digits of mobile telephone numbers) that do not exist.

The sample was obtained, using stratified random sampling by size of habitat and Autonomous Region (Comunidad Autónoma), with homes as first-stage sampling units. The sizes of the strata were obtained a priori on the basis of official statistics of the Spanish adult population, and the persons to be interviewed in each stratum were selected by simple random sampling with poststratification by sex and age group, until the pre-established sample size in each stratum was reached. This task was automatically performed with the aid of Bellview CATI (computer-aided telephone interview) computer software. The interview was designed to obtain a 95.5% confidence level, with a precision of ±3.5% for an estimated proportion of 50%. The response rate was 76%, thus making it necessary to select a total of 1319 individuals until the pre-established sample size was reached. The final sample totaled 1002 participants with proportional allocation per stratum.

### 2.2. Data Collection and Study Variables

The study questionnaire was purpose-designed by the study researchers taking other questionnaires used in similar interviews as reference [6,8,9,10,15,25,26,27,28,29,30], and then sent to public health policy experts and representatives of the Food and Agriculture Organization (FAO) and the WHO, whose suggestions were subsequently incorporated. To ascertain the appropriateness, comprehensibility and order of the questions, the length and duration of the questionnaire, and the level of response, we carried out a pilot study on a sample of 60 persons from 30 May to 6 June 2018. Due to difficulties of comprehension or inconsistencies in responses, the wording of 3 questions was amended halfway through the field work of this pilot study.

The study questionnaire was made up of 40 questions structured in 4 sections. The dependent variable of the study was a question using a 5-point Likert-type scale (“strongly agree”, “agree”, “no opinion”, “disagree”, and “strongly disagree”) to evaluate support for a tax on sugar-sweetened beverage, which formed part of the section on price policies. We considered tax supporters as those who responded they agree or strongly agree. In view of the fact that the pilot study showed that support for this measure varied according to the precise order in which the questions on price policies were posed, a random order was applied, with half of the participants first being asked about the tax, and the other half first being asked about reductions in VAT and subsidies on healthy products. Using the same Likert-type scale, we assessed the degree to which participants agreed with the attribution of obesity to different causes. The section on health included questions on weight and height, physical activity, sleep, and food. Lastly, we included a section addressing sociodemographic information, with data on sex, age, nationality, educational level, marital status, occupational status, income level, political orientation, and occupation, which served to assign social class using the classification of occupational social class (CSO-SEE12), based on the Spanish National Classification of Occupations 2011 (CNO-2011) and a neo-Weberian Perspective [41]. The interviews were conducted from 10 September to 1 October 2018, using a CATI having a mean duration of 20 min, administered by trained interviewers.

### 2.3. Statistical Analysis

We performed a descriptive analysis by calculating the distribution of the frequencies and, where applicable, the mean of the sample’s sociodemographic characteristics, consumption of sugar-sweetened beverages, presence of excess weight (body mass index (BMI) >25), and level of attribution of obesity to different causes. The degree of support for the tax on sugar-sweetened beverages was determined by calculating the percentage of those who agreed plus those who strongly agreed with the measure. To compare support for the tax by category of study variable and the order in which the question was posed (i.e., before or after asking about tax relief and subsidies on healthy foods), the chi-squared test (χ^2^) was applied. To analyze the determinants of support for the tax, we used Poisson regression models with robust variance, adjusted for consumption of sugar-sweetened beverages, BMI, and sociodemographic variables identified as relevant in the scientific literature [27,28,29,30,31]. Poisson regression with robust variance provides correct estimates and is a better alternative to logistic regression for the analysis of cross-sectional studies with binary outcomes [42]. As the results showed no variation after we excluded the variables of ideological orientation and income level, which displayed a high number of missing values, the models are shown without adjustment for these variables. To correct small deviations in the final valid sample with respect to the proportional allocation, in all calculations we applied a weighting coefficient for each case, having regard to the proportional distribution of the Spanish population by the variables of sex, age, autonomous region, and habitat.

## 3. Results

### 3.1. Sociodemographic Characteristics

The sociodemographic characteristics of the sample are shown in Table 1. The mean age of the 1002 participants was 50.3 years, and 52.7% were women. The breakdown showed the following: more than half had secondary education or higher (57.5%), were gainfully employed (53.2%), ideologically aligned with the political center (50.4%), and reported an income of less than EUR 1850 (57.5%); 42.4% were classified as having low social class status, 43.3% suffered from excess weight, and 22.5% were regular consumers of sugar-sweetened beverages.

### 3.2. Degree of Agreement with Attribution of Obesity to Individual and Environmental Causes

Participants “agreed” or ”strongly agreed” with obesity being essentially attributed to individual factors, with 92.2% attributing it to lack of effort, motivation, and discipline, 95.4% attributing it to addiction to food high in fat, sugar, or salt, and 97.2% attributing it to consumption of foods and sugar-sweetened beverages, as opposed to 75.7% who attributed it to genetics. Attribution of obesity to environmental factors was 69.9% for the high price of healthy foods and 86.8% for the low price of unhealthy foods. On a scale of 1 (strongly disagree) to 5 (strongly agree), mean scores ranged from 4.6 for excessive consumption of foods and sugar-sweetened beverages to 3.8 for genetics and the high price of healthy foods (Table 2).

### 3.3. Prevalence of Support for a Tax on Sugar-Sweetened Beverages

A total of 66.9% of participants supported the imposition of a tax on sugar-sweetened beverages (Table 3). The level of support for the tax was lower among participants professing a right-wing ideological orientation (51.2%; *p* < 0.01), persons who reported an income below EUR 1050 (61.2%; *p* = 0.04), those having low social class status (62.7%; *p* = 0.04), and regular consumers of sugar-sweetened beverages (57.2%; *p* < 0.01). The level of support for the tax was higher among participants who strongly agreed with obesity being attributed to excessive consumption of sugar-sweetened beverages, addiction to sugar, and lack of motivation and discipline (71%; *p* < 0.01); the high price of healthy foods (74.6%; *p* < 0.01); the low price of unhealthy foods (72.2%; *p* < 0.01; data not shown in the Table). The level of support for the tax was 9.2% higher (*p* = 0.049) in those cases where participants were asked about it after being asked about support for tax relief and subsidies for healthy foods (Table 3). This difference was more marked among women (13.1%; *p* = 0.04), adolescents and young adults (27.8%; *p* = 0.05), university students (18.5%; *p* = 0.04), participants ideologically aligned with the political center (17.6%; *p* = 0.03), middle-class participants (20.4%; *p* = 0.02), and those who suffered from overweight (15.6%; *p* = 0.04).

### 3.4. Prevalence Ratios of Support for a Tax on Sugar-Sweetened Beverages

Table 4 shows the prevalence ratios (PRs) of support for the tax obtained with Poisson regression models. In comparison with participants who professed a left-wing ideological orientation, support for the tax was 18% and 34% lower among those who reported center-leaning (PR = 0.82; CI: 0.75–0.91) and right-wing political affiliations, respectively (PR = 0.66; CI: 0.54–0.81). Support for the tax was also 15% lower among persons with a monthly income of under EUR 1050 (PR = 0.85; CI: 0.75–0.97) as compared to those who reported an income of over EUR 1850, and 18% lower among regular consumers versus nonconsumers of sugar-sweetened beverages (PR = 0.82; CI: 0.73–0.93). Although these effects remained unchanged in the adjusted models, the effect for income ceased to be statistically significant.

## 4. Discussion

The practical totality of the Spanish population (97.2%) feels that excessive consumption of sugar-sweetened beverages causes obesity, and two out of every three people are in favor of a tax on sugar-sweetened beverages, a level of support that drops among regular consumers of sugar-sweetened beverages and those who profess center-leaning or right-wing ideological affiliations. Individuals who attribute obesity to consumption of sugar-sweetened beverages and the factors underlying this are more strongly in favor of the measure. Support for the tax rose by 9.2% when participants were asked about it after other price measures, such as subsidies or tax cuts on healthy products, had been proposed, with this figure doubling among university students and middle-class persons.

This is the first study to analyze the opinions of a representative sample of the Spanish adult population about the imposition of a tax on sugar-sweetened beverages. The proposed tax received majority support (66.9% of the sample), higher than that observed in countries such as France (57.7%) [43], the USA (40%) [38], Germany (42.2%) [37], and Australia (48%) [44]. Only one recent survey in the United Kingdom (UK) showed itself more favorable to this type of tax, with 70% in support [45], though at the time the UK survey was carried out, the tax had already been announced by the Government, and support for such measures is known to increase after their implementation [25]. In Catalonia, the only Autonomous Region in which the tax has been introduced [15], the level of support also reached 70%, though the difference with respect to the rest of Spain was not statistically significant (data not shown in the tables). Furthermore, in the UK, the measure is targeted at drink manufacturers and the tax revenues will be devoted to health promotion activities, aspects that are linked to a higher level of support [36,43,44,46,47]. Our data support this thesis because, when participants were consulted about other types of price measures, such as tax relief and subsidies on healthy foods, before being asked about the tax, support rose to 70%, equaling that of the UK. This leap was particularly marked among people professing ideological alignment with the political center and right, and among teenagers and young adults (ages 18–29 years), as was the case in Australia [44]. In addition to enjoying more popular support, price policies that combine taxes with subsidies are not only more effective [48,49], but also mitigate the possible negative economic impacts of such taxes on the most underprivileged classes [50]. At all events, a recent review found that taxation measures have neutral or positive impacts on social inequalities in obesity and obesity-related habits, such as consumption of sugar-sweetened beverages [51].

A total of 97.2% of participants attributed obesity to consumption of sugar-sweetened beverages, a figure higher than that of around 90% observed in other countries [31,33,45], something that would help to explain the high level of support for the tax in Spain [25]. The participants overwhelmingly agreed that obesity is due to causes linked to consumption of sugar-sweetened beverages, the responsibility for which must be attributed to the individual. This result is in line with a narrative that suggests that obesity is essentially a question of individual responsibility [52], a way of perceiving and addressing the dominant problem in Spain and other countries in the region [26,53], reinforced and boosted by the mass media [54], which prioritizes the application of measures designed to act on the individual rather than the environment [35]. In such a context, it is no surprise that support for the tax would in great part depend on political ideology, with less backing from participants who profess center-leaning and right-wing political affiliations, a finding in line with the results of other studies [29,31,33,38,55,56].

As in the UK [45] and Australia [44], support for the tax was lower among regular consumers of sugar-sweetened beverages, the main segment affected by the measure, given that there is a tendency for greater support to be given to restrictive interventions aimed at the behavior patterns of others rather than of oneself [25]. The differences observed by social class and income level (less support for the tax at the lowest levels), a finding in line with the results of other [35] (though not all) [47] studies, disappeared when adjustment was made for other sociodemographic variables. Similarly, no differences were found by age or educational level, which were indeed observed in other [30,31,38] (though, yet again, not all) [43] studies. As in other studies, attribution of obesity to the consumption of sugar-sweetened beverages and addiction to food was linked to greater support for the tax [30,33,45,56]. Backing for the tax was also higher among those who attributed obesity to environmental causes, such as the price of foods, a finding along similar lines to that observed in the US [38] and German populations [37].

### Limitations

The principal limitation of the study involves a possible nonresponse bias, in that 24% of the individuals selected refused to participate. As a bias-correction technique, we used semicontrolled sampling with replacement, whereby corrected prevalence estimates were obtained that are similar to those used with other methods [57]. Although this technique ensured that the sample continued to be representative of the Spanish population in terms of the sociodemographic characteristics used in the sampling, it is nevertheless possible that the individuals who were most motivated to respond might differ from those who did not respond in terms of other characteristics that determine support for the tax. A further limitation relates to the size of the sample, which was large enough for estimating the prevalence of support for the tax but limited for studying small-magnitude associations with sufficient precision. Moreover, the fact that this was a cross-sectional study means that the causality of the associations observed cannot be established. Other possible biases are social desirability bias and, in the case of the question regarding the consumption of sugar-sweetened beverages, recall bias. Although social desirability has been shown to influence reported sweetened beverage beliefs about the economic benefits of taxes in opinion surveys [58], the support for the tax is so high in Spain that the magnitude of the potential bias could not alter the conclusions of the study. In addition, weight and height were self-reported, something that tends to give rise to an underestimate of BMI. Lastly, later events since the data collection may have shifted Spanish consumer attitudes. However, even the disruptive COVID-19 pandemic had no impact on the consumption of sugar-sweetened beverages among adolescents, the main market for these drinks [59], and in Catalonia, where the tax has been implemented since 2017, there were no changes in the upward trend of the tax’s effect either during or after confinement [60].

## 5. Conclusions

In view of our results, the imposition of a tax on sugar-sweetened beverages to discourage their consumption, a WHO-recommended intervention of proven effectiveness, would be well received by the Spanish population, something that would facilitate its implementation and success. To increase support for the tax and mitigate its possible undesired effects, it would be advisable to combine the measure with subsidies or tax cuts on healthy foods, and with awareness-raising campaigns about the causes of obesity, environmental and genetic, which are not within the scope of individual control.

## Figures and Tables

**Table 1:** Sociodemographic characteristics of the study sample, representative of the Spanish adult population: 2018.

Sociodemographic Characteristics	*n*	%
Total	1002	100
Sex		
Men	474	47.3
Women	528	52.7
Age (years)		
>65	240	23.9
45–64	358	35.7
30–44	251	25.0
18–29	153	15.3
Educational level		
University	271	27.0
Secondary	576	57.5
Primary	155	15.5
Occupational status (*n* = 1001)		
Gainfully employed	533	53.2
Pensioner	255	25.4
Unemployed/unremunerated work	146	14.6
Student	67	6.7
Ideology (*n* = 855)		
Left-wing	319	37.3
Center	431	50.4
Right-wing	105	12.3
Income (*n* = 794)		
>EUR 1850	337	42.4
EUR 1050–1850	251	31.6
<EUR 1050	206	25.9
Social class (*n* = 922)		
High	273	29.6
Middle	258	28.0
Low	391	42.4
BMI * (*n* = 986)		
<25	559	56.7
>25	427	43.3
Consumption of sugar-sweetened drinks		
Nonconsumer	655	65.4
Occasional (1 a week)	121	12.1
Regular (>1 a week)	226	22.5

* BMI: body mass index.

**Table 2:** Degree of agreement with attribution of obesity to individual and environmental causes.

Attribution of Obesity	*n*	Strongly Disagree (%)	Disagree (%)	No Opinion (%)	Agree (%)	Strongly Agree (%)	Mean * (SD)
Individual causes							
Excessive consumption of foods and sugar-sweetened beverages	1000	0.5	1.3	1	32.3	64.9	4.6 (0.63)
Addiction to food high in fat, sugar, or salt	994	0.4	2	2.2	35.9	59.5	4.5 (0.68)
Lack of effort, motivation, and discipline among people who suffer from obesity	990	0.5	2.4	4.8	42.1	50.1	4.4 (0.73)
Genetics	938	1.2	10.4	12.6	54.3	21.3	3.8 (0.92)
Environmental causes							
High price of healthy foods	986	2.4	16.6	11.8	36.2	32.9	3.8 (1.14)
Low price of unhealthy foods	998	0.9	6.9	5.4	36.1	50.7	4.3 (0.91)

* Scale: 1–5 (1 “strongly disagree”; 2 “disagree”; 3 “no opinion”; 4 “agree”; 5 “strongly agree”).

**Table 3:** Prevalence of support for a tax on sugar-sweetened beverages, overall and by order of questions on price policies.

	Overall (N = 993)	*p*	By Question Order		Relative Difference ** %	*p*
			Taxes First (N = 517)	Subsidies and Tax Relief First (N = 485)		
Total	66.9		64.1	70.0	9.2	0.05
Sex		0.30				
Men	65.3		63.8	67.0	5.1	0.47
Women	68.4		64.3	72.8	13.1	0.04
Age (years)		0.53				
>65	67.5		63.3	72.2	14.0	0.16
45–64	69.5		69.0	70.0	1.3	0.85
30–44	65.2		63.1	67.7	7.4	0.45
18–29	63.4		55.6	71.0	27.8	0.05
Educational level		0.14				
University	69.9		63.9	75.7	18.5	0.04
Secondary	67.2		64.6	70.2	8.8	0.16
Primary	60.4		62.4	58.0	−7.2	0.58
Occupational status (*n* = 992)		0.56				
Gainfully employed	66.8		64.5	69.1	7.0	0.28
Pensioner	65.9		62.0	70.2	13.3	0.18
Unemployed/unremunerated work	71.3		66.4	76.7	15.6	0.17
Student	62.1		62.1	62.1	0.0	1
Ideology (*n* = 849)		<0.01				
Left-wing	77.1		77.0	77.3	0.4	0.95
Center	63.6		58.7	69.0	17.6	0.03
Right-wing	51.2		45.9	58.2	26.9	0.23
Income (*n* = 788)		0.04				
>EUR 1850	71.7		69.1	73.9	7.0	0.34
EUR 1050–1850	68.9		66.7	72.3	8.4	0.36
<EUR 1050	61.2		56.8	66.3	16.8	0.17
Social class (*n* = 915)		0.04				
High	69		66.2	71.5	7.9	0.36
Middle	72		65.9	79.3	20.4	0.02
Low	62.7		62.1	63.3	1.9	0.81
BMI * (*n* = 978)		0.42				
<25	68.2		66.7	69.8	4.6	0.44
>25	65.8		61.4	71.0	15.6	0.04
Consumption of sugar-sweetened beverages		<0.01				
Nonconsumer	69.4		66.2	73.1	−10.4	0.06
Occasional (1 a week)	72.0		70.1	74.2	−5.8	0.62
Regular (>1 a week)	57.2		54.4	60.0	−10.3	0.40

* BMI: body mass index. ** Relative difference was calculated: [(support when asked about subsidies and tax relief first − support when asked about taxes first)]/(support when asked about taxes first).

**Table 4:** Prevalence ratios (95% CI) of support for a tax on sugar-sweetened beverages obtained with Poisson regression models.

	Crude Model	*p*	Adjusted Model *	*p*
Sex		0.3		0.76
Men	1		1	
Women	1.05 (0.96–1.14)		1.01 (0.92–1.12)	
Age (years)		0.53		0.76
>65	1		1	
45–64	1.03 (0.92–1.15)		0.97 (0.83–1.14)	
30–44	0.96 (0.85–1.1)		0.92 (0.76–1.11)	
18–29	0.94 (0.81–1.09)		0.93 (0.74–1.17)	
Educational level		0.17		0.36
University	1		1	
Secondary	0.96 (0.87–1.06)		0.98 (0.87–1.09)	
Primary	0.86 (0.74–1.00)		0.87 (0.72–1.06)	
Occupational status		0.54		0.23
Gainfully employed	1		1	
Pensioner	0.99 (0.88–1.10)		0.98 (0.83–1.15)	
Unemployed/unremunerated work	1.07 (0.95–1.20)		1.11 (0.97–1.27)	
Student	0.93 (0.76–1.13)		1.21 (0.93–1.59)	
Ideology		<0.01		<0.01
Left-wing	1		1	
Center	0.82 (0.75–0.91)		0.84 (0.77–0.93)	
Right-wing	0.66 (0.54–0.81)		0.65 (0.53–0.81)	
Income		0.06		0.17
>EUR 1850	1		1	
EUR 1050–1850	0.96 (0.86–1.07)		1 (0.88–1.11)	
<EUR 1050	0.85 (0.75–0.97)		0.87 (0.74–1.01)	
Social class		0.04		0.17
High	1		1	
Middle	1.04 (0.93–1.17)		1.06 (0.94–1.19)	
Low	0.91 (0.81–1.02)		0.95 (0.83–1.08)	
BMI ^#^		0.43		0.9
<25	1		1	
>25	0.96 (0.88–1.05)		0.99 (0.90–1.09)	
Consumer of sugar-sweetened beverages		<0.01		0.01
Nonconsumer	1		1	
Occasional	1.04 (0.91–1.17)		1.06 (0.93–1.21)	
Regular	0.82 (0.73–0.93)		0.84 (0.73–0.96)	

* Adjusted for all variables in the table except ideology and income. **^#^** BMI: body mass index.

## Data Availability

The datasets used during the current study are available from the corresponding author on reasonable request at mroyo@isciii.es.
